# Mechanical Dilution of Beta-amyloid Peptide and Phosphorylated Tau Protein in Alzheimer's Disease: Too Simple to be True?

**DOI:** 10.7759/cureus.1062

**Published:** 2017-02-28

**Authors:** Manuel Menéndez González

**Affiliations:** 1 Neurology, Hospital Universitario Central de Asturias

**Keywords:** csf derivation, amyloid beta, tau protein, tau phosphorylation, csf, alzheimer disease, mild cognitive impairment, neurodegenerative disease

## Abstract

The neuropathology of Alzheimer's disease (AD) is characterized by the widespread accumulation of neuritic plaques and neurofibrillary tangles composed of deposits of beta-amyloid peptide (Aβ) and abnormally phosphorylated tau protein (phospho-tau) respectively. Considerable effort has been expended to identify methods to retard the deposition of these proteins or to enhance their clearance. It is strikingly surprising that until now, very few researchers have attempted to remove these proteins using mechanical procedures.

In this article, we start by showing the rationale of mechanical dilution of cerebrospinal fluid (CSF) as a therapeutic approach in AD. Then, we present models of implantable systems allowing mechanical dilution of CSF by means of CSF replacement and CSF filtration (liquorpheresis). We conclude that even though this approach seems simplistic, it is feasible and deserves exploration.

## Introduction

### Rationale for mechanical dilution of cerebrospinal fluid (CSF) in Alzheimer's disease (AD)

**Figure 1:**
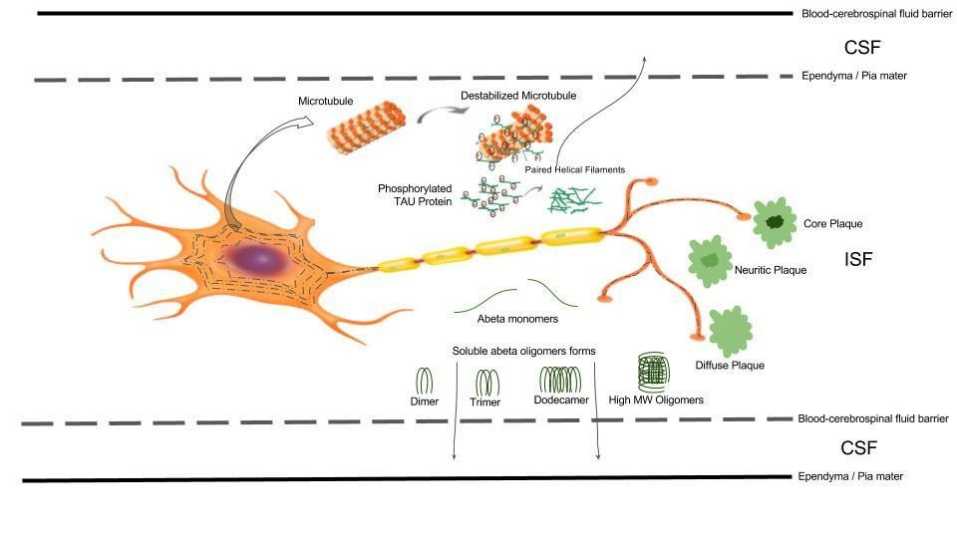
Aβ and phospho-tau from neurons to CSF Schematic drawing of a neuron with dendrites to the left and the axon and synaptic tree to the right. Up: neurofibrillary tangles composed of hyperphosphorylated tau, which are characteristic of AD, are present in the cell body and are shown in discontinuous black lines. The curved arrow pointing from this line shows a zoom of hyperphosphorylated tau. Both tau and phospho-tau exist from ISF to the CSF. Down, in the ISF, soluble Ab monomers exist in equilibrium with small Ab oligomers (dimers, trimers and dodecamers) and high-molecular-weight (MW) aggregates, including diffuse and more mature forms of the Ab plaques that are depicted in the right part of the panel. Soluble Ab monomers and smaller oligomers and aggregates may also diffuse to the CSF.

The neuropathology of AD is characterized by the widespread accumulation of neuritic plaques and neurofibrillary tangles composed of deposits of beta-amyloid peptide (Aβ) and abnormally phosphorylated tau protein (phospho-tau) respectively (Figure 1). Aβ accumulation has been hypothesized to result from an imbalance between Aβ production and clearance. Indeed, Aβ clearance seems to be impaired in both early and late forms of AD. Soluble Aβ can be removed from the brain by various clearance systems including enzymatic degradation and cellular uptake, transport across the blood-brain barrier (BBB) and blood-cerebrospinal fluid barrier (BCSFB), interstitial fluid (ISF) bulk flow, and CSF absorption into the circulatory and lymphatic systems. The meningeal lymphatic vessels, recently discovered, might provide another clearance route. Since these clearance systems act together to drive Aβ from the brain, any alteration to their function could contribute to AD. Besides this, tau protein becomes hyperphosphorylated (phospho-tau) in AD getting unstable and unable to bind the microtubules and finally disintegrating into neurofibrillary tangles. Specific BBB transporters for tau have not been identified. This suggests that clearance of tau is less complex than that of Aβ, and mainly relies on degradation, CSF absorption, and ISF bulk flow [1].

 

Considerable effort has been expended to identify methods to retard the deposition of these proteins or to enhance their clearance [2]. Perhaps the most common therapeutic approach being explored today is the clearance of Aβ from the brain by activating the immune system against Aβ (Aβ immunotherapy), and against tau (tau immunotherapy). Thus, Alzheimer immunotherapies consist of the administration (passive immunotherapy) or induction (active immunotherapy) of anti-Aβ or anti-tau antibodies [3]. Regrettably, all clinical trials have failed to date. Something that we have all learned from trials is that any treatment in AD should be addressed at the very early stages of the disease. Otherwise, it will be useless even when some neuropathological changes can be achieved. This is probably because by the time the neuritic plaques and neurofibrillary tangles are in place, much neuronal damage has already been done. Then, we need to remove phospho-tau and soluble Aβ before they get deposited (Figure 1).

It is strikingly surprising that until now, very few researchers have attempted to remove these proteins using mechanical procedures. There are a good number of studies where AD CSF biomarkers were correlated with prognosis of long-term cognitive outcomes in patients with idiopathic normal pressure hydrocephalus (iNPH) after shunting treatment. It is important to note that in these studies, AD comorbidity could not be excluded before shunting. Indeed, cortical biopsy findings indicate that normal pressure hydrocephalus (NPH) is at present a heterogeneous syndrome and has notable overlapping with AD [4-11]. In most of these studies, authors found a lack of shunt response in suspected iNPH with AD pathology. In one of these studies, authors found that high p-tau/Aβ1-42 ratios in ventricular CSF correlated with the presence of cortical AD pathology. At baseline, iNPH patients with ratio values most suggestive of AD presented with better gait performance but poorer cognitive performance. Patients with high p-tau/Aβ1-42 ratios also showed a less robust response to shunting on both gait and cognitive measures. Finally, in a subset of 18 patients who also underwent lumbar puncture, ventricular CSF ratios were significantly correlated with lumbar CSF ratios [9]. In a second study, the p-tau Aβ1-38/Aβ1-42, and Aβ1-42/p-tau ratios predicted prognosis of cognitive function, with worse prognosis in those with a basal AD profile [10]. Analysis of the Aβ1-38/Aβ1-42 ratio revealed that, while the “Improved cognitive group” showed a tendency to shift from Aβ1-42 to Aβ1-38 two years after surgery, the “Poor cognitive group” showed similar results before and two years after the same. The switch in Aβ-variant synthesis from Aβ1-42 to Aβ1-38 also resulted in the improvement of functional prognosis. Interestingly, concentrations of Aβ1-42 (and Aβ1-38) increased after shunt treatment in both groups. Authors of the second study suggest that improved CSF circulation in iNPH after LPS increases the amount of cystatin C and L-PGDS, possibly contributing to Aβ elimination and improvement of a range of symptoms [10].

There is a theory where AD is proposed to be directly caused by a CSF circulatory failure [12-13]; the initially dominant physiological change determines whether the CSF circulatory failure manifests as AD or as iNPH. If CSF production failure predominates, AD develops. However, if resistance to CSF outflow predominates, it results in NPH. The authors of this theory have also studied the safety and efficacy of improving CSF turnover in 29 patients (aged 62-85 years) with mild to moderate AD (NINDS-ADRDA criteria) by means of a novel, constant, and low-flow ventriculoperitoneal shunt (COGNIShunt, Redwood City, CA, USA). The COGNIShunt system was evaluated under an FDA investigational device exemption (IDE). There were no unexpected adverse events when compared with a similar population of elderly patients given shunts for iNPH. Particularly, no subject had symptoms of over drainage, the side effect the shunt was specifically designed to avoid. Based on the encouraging preliminary data, COGNIShunt entered into a pivotal study that eventually recruited 215 patients at 22 sites. According to a press release by the owner of COGNIShunt [14], enrollment to the study was halted by the company in December 2003 based on the results of a planned interim analysis. The analysis indicated that the new sample size estimation was greater than the stopping rule number previously agreed upon with the FDA. Although the analysis of the more sensitive measure of mental function demonstrated a difference between the two groups in favor of the COGNIShunt group, analysis of the global deterioration scale (GDS) did not show a difference; therefore, the pivotal study, as designed, would not have been sufficient to demonstrate efficacy in support of a US premarket approval application (PMA). All implanted subjects continued with per-protocol study procedures while the company continued to analyze the endpoint measures data. In June 2004, the study was closed based on the results of the second interim analysis which showed that the difference between treatment groups, while still favoring the COGNIShunt group, was less than that of the first interim analysis [13].

Putting aside the theory conceiving AD as a CSF circulatory failure, there is no doubt Aβ and phospho-tau play a central role in the pathogenesis of AD, and removing them would be a major step towards the cure of the disease. All previous studies used the classical one-way ventriculo-peritoneal shunt, where very limited clearance of CSF peptides takes place by a diluting effect. We propose a new approach for mechanical clearance of Aβ and phospho-tau by diluting their concentration in the CSF continuously. This can be achieved by using implantable systems for CSF replacement or CSF filtration.

## Technical report

### Implantable systems for CSF mechanical dilution

The CSF volume, estimated to be about 150 ml in adults, is distributed between 125 ml in cranial and spinal subarachnoid spaces and 25 ml in the ventricles, but with marked interindividual variations. CSF production is around 500 ml per day and it is renewed about four times every 24 hours [13, 15]. As told, reduction of the CSF turnover rate has been described in aging and in AD [13-14].

**Figure 2:**
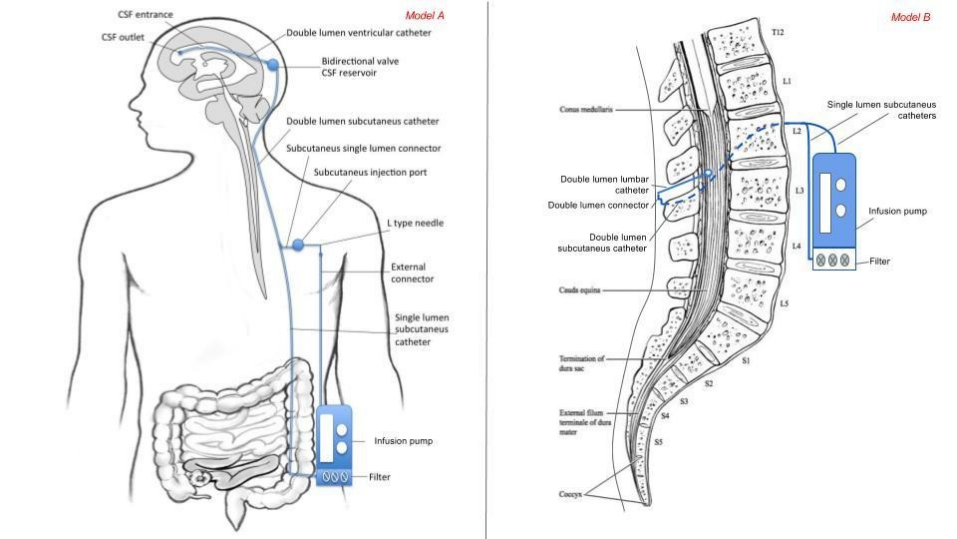
Implantable liquorpheresis systems Model A, on the left: ventricular liquorpheresis. Model B, on the right: lumbar liquorpheresis.

Some implantable systems for CSF replacement and CSF filtration (liquorpheresis) have been described [16]. The central catheter of these systems may be placed in the ventricular system (ventricular shunting) or in the lumbar subarachnoid space (intrathecal shunting). Implanting these systems require a surgical procedure very similar to that used for the placement of valves, ventriculo-peritoneal or lumbar-peritoneal derivation. Key elements of implantable systems for liquorpheresis are represented in Figure 2.

**Figure 3:**
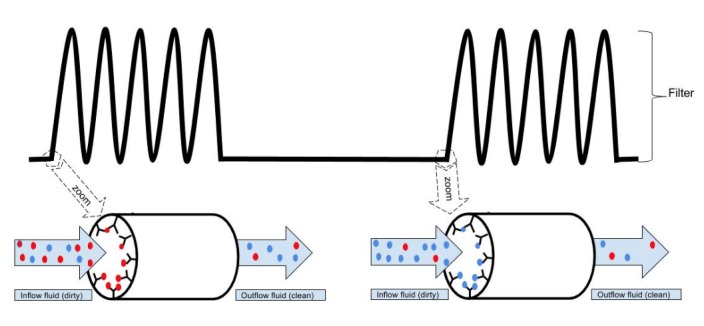
Schematic representation of an immunotechnological filter Red balls represent Aβ and blue balls represent tau. The inside of a long column is fully coated with antibodies against the target molecules in a sequential mode. In the first portion of the filter, the column is coated with antibodies against Aβ and in the second portion with antibodies against tau. As the fluid passes through the filter, the target molecules get stuck to the antibodies. As a result, the concentration of Aβ and tau is much lower in the outflow fluid than in the inflow fluid.

Several different types of filters for Aβ and tau can be developed in the future; from mechanical filters, similar to those used in plasmapheresis, to "immunotechnological filters", based on antibodies against Aβ and tau able to catch these proteins from the CSF (Figure 3). Biological filters of CSF may represent a highly innovative field of research combining nanotechnology with immunotechnology (i.e., magnetic nanoparticles conjugated with antibodies).

## Discussion

These systems allow the replacement of “dirty” CSF (with Aβ and phospho-tau) with “clean” CSF (without Aβ and phospho-tau) several times a day. With any of these systems, the CSF concentration of Aβ and phospho-tau should decrease sharply. This decrease is supposed to have a “clearance effect” over the ISF concentration of Aβ and phospho-tau (Table 1) [1], preventing their deposition.

**Table 1:** CSF dynamics Facts of CSF dynamics in normal subjects and hypothetical effects of mechanical dilution of Aβ and phospho-tau in AD.

	Normal	Untreated AD	Treated AD
CSF production	～500 ml/day	< 500 ml/day?	?
CSF volume	～150 ml	～150 ml	～150 ml
Renewal rate	～4 times/day	<4 times/day ?	>4 times/day
CSF Aβ concentrations		↓	↓↓
CSF phospho-tau concentrations		↑	↓↓
ISF Aβ concentrations		↑	↓
ISF phospho-tau concentrations		↑	↓

We cannot compute the extent of this “clearance” effect” because there are some variables we still do not know about. For instance, we do not know how such systems would affect the production of CSF. Anyway, the dilutional effect would be particularly driven by the renewal rate and how the turnover is programmed.

## Conclusions

We show the rationale for mechanical dilution of CSF as a therapeutic approach in AD. Mechanical dilution of Aβ and phospho-tau at early stages of the disease may ease clearance of these peptides in the interstitial fluid. Thus, we can prevent the deposition of these peptides in the brain tissue. We also review some innovative models of implantable systems for CSF replacement and liquorpheresis.

The idea of treating AD by simply diluting "dirty" CSF with "clean" CSF may seem far-fetched at first. However, from a theoretical point of view, this seems a completely feasible approach that deserves exploration. Product development and preclinical studies should be addressed to verify the usefulness of these systems.
